# MicroRNAs as Predictor Markers for Response to Interferon Treatment of Chronic Hepatitis C Genotype-4 in Egyptian Patients

**DOI:** 10.1371/journal.pone.0121524

**Published:** 2015-03-26

**Authors:** Tarek M. K. Motawi, Sherine M. Rizk, Olfat G. Shaker, Olfat Z. H. Mokhtar

**Affiliations:** 1 Department of Biochemistry, Faculty of Pharmacy, Cairo University, Cairo, Egypt; 2 Department of Medical Biochemistry, Faculty of Medicine, Cairo University, Cairo, Egypt; 3 New Educational El kasr El Aini Hospital, Cairo University, Cairo, Egypt; University of Sydney, AUSTRALIA

## Abstract

**Background:**

Hepatitis C virus genotype 4 (HCV-4) infection is common in the Middle East and Africa, with an extraordinarily high prevalence in Egypt. MicroRNAs (miRNAs) play an important role in various diseases, including HCV infection. The aim of the present study was to assess serum miR-122, miR-221 and miR-21 expression profiles in HCV-4 patients prior to treatment with HCV-4 combination therapy (pegylated alpha interferon and ribavirin) and to determine whether the miRNAs were associated with the drug response.

**Methods:**

RNA was extracted from pretreatment serum samples, and miR-122, miR-221 and miR-21 levels were measured by quantitative PCR. The results were compared among patients with sustained virological responses (SVR) and non-responders (NR).

**Results:**

The expression levels of miR-21 and miR-122 were significantly different between the SVR and NR groups. Receiver operator characteristic (ROC) analysis revealed that the sensitivity, specificity and positive predictive values of miR-21 were 82.2%, 77.3% and 88.1%, respectively, with a cut-off value of 1.7. The sensitivity, specificity and positive predictive values of miR-122 were 68.9%, 59.1% and 77.5%, respectively, with a cut-off value of 3.5.

**Conclusion and Significance:**

miR-21 and miR-122 might be useful predictors for SVR in HCV-4 patients prior to the administration of combination therapy. A higher predictive response power was obtained for miR-21 than for miR-122. These results should reduce ineffective treatments.

## Introduction

Hepatitis C virus (HCV) is one of the risk factors for liver disease [1]. HCV has been classified into seven major genotypes and a series of subtypes [2, 3]. In general, HCV genotype 4 (HCV-4) is common in the Middle East and Africa, where it is responsible for more than 80% of HCV infections [4]. Egypt has the highest prevalence of HCV infection worldwide (15%) and the highest prevalence of HCV-4; HCV-4 is responsible for 90% of the total HCV infections in Egypt, with a predominance of subtype 4a (HCV-4a) [5]. This extraordinarily high prevalence has resulted in an increasing incidence of hepatocellular carcinoma in Egypt, which is now the second most frequent cause of cancer and cancer mortality among men [6]. More than two decades have passed since the discovery of HCV, and yet therapeutic options remain limited. Up to 2011, the standard treatment for chronic hepatitis C consisted of a combination of pegylated alpha interferon (PEG-IFN) and ribavirin (RBV) [7]. The response of HCV-4 to the standard regimen of treatment (PEG-IFN/RBV) has lagged behind other genotypes, and HCV-4 has become the most resistant genotype to treatment. Because PEG-IFN/RBV continue to be used to treat HCV-4-infected patients, an exploration of the factors that predict the outcome of PEG-IFN/RBV treatment (i.e., sustained virological response (SVR)) for HCV-4 infection is needed to more accurately assess the likelihood of SVR, and thus to enable more informed treatment decisions.

MicroRNAs (miRNAs) are a group of small noncoding functional RNAs that are approximately 22 nucleotides in length [8]. miRNAs play pivotal roles in most critical biological events, including development, proliferation, differentiation, cell fate determination, apoptosis, signal transduction, organ development, hematopoietic lineage differentiation, host-viral interactions and tumourigenesis [9, 10].

Viruses use miRNAs in their efforts to control their host cell; reciprocally, host cells use miRNAs to target essential viral functions. Experimental results have shown that miRNAs are involved in innate immunity and function as both gene regulators and a host cell defence against RNA and DNA viruses [11, 12]. For example, the expression of host cell miR-122 can inhibit the replication of HCV through IFN-β [13].

The liver-expressed miR-122 is essential for HCV RNA accumulation in cultured liver cells, but its potential as a target for antiviral intervention has not been assessed [14]. miR-122 has a positive role in HCV replication. The HCV genome contains 4 binding sites for miR-122. miR-122 has been implicated in the regulation of different metabolic pathways in liver cells (i.e., cholesterol metabolism), and has become the most studied miRNA involved in HCV infection [15]. The role of miR-122 in HCV RNA replication was confirmed by silencing miR-122 in Huh7 cells, resulting in a marked loss of replication. miR-122 stimulates HCV translation by enhancing the association of ribosomes with the viral RNA at an early initiation stage; its expression was found to be significantly down regulated in tumours compared with non-malignant liver tissues [16].

miR-221 is a popular example of a miRNA that is critical for hepatocellular carcinoma (HCC) development due to apoptosis regulation. The overexpression of miR-221 was shown to lead to transcriptional induction of two cyclin-dependent kinase inhibitors (CDKIs) (CDKN1C/p57 and CDKN1B/p27) that support the cell proliferation of hepatocytes. Indeed, these two CDKNIs were shown to serve as respective targets for miR-221 during the course of HCC development in humans [17].

miR-21 is linked to human liver pathogenesis, ranging from normal liver integrity to cirrhosis to HCC. The expression of this miRNA has been used as an example of the relevance of specific miRNAs to disease progression, starting from the induction of hepatitis to liver cirrhosis and finally to HCC [17].

To date, very limited data exists concerning the changes in expression patterns of miR-122, miR-221 and miR-21 in response to drugs in HCV-4 patients. In light of this deficiency, the aim of the present study was to evaluate miR-122, miR-221 and miR-21 expression profiles in HCV-4 patients prior to treatment with HCV-4 combination therapy and to determine whether the miRNAs were associated with the drug response. Knowledge of patients’ expression profiles is expected to provide a clear understanding of how aberrant expression of miRNAs can aid in the development of more effective and safer therapeutic strategies for HCV-4.

## Subjects and Methods

### Study Design

Inclusion Criteria: patients with elevated alanine aminotransferase (ALT) and aspartate aminotransferase (AST) levels (>37 IU/L) who were positive for HCV antibodies and RNA viral loads (HCV-RNA genotype-4) within 6 months prior to the study were enrolled. Exclusion Criteria: HCV infected patients under the age of 20 and over the age of 55 years, co-infected with hepatitis B virus (HBV) or human immunodeficiency virus (HIV), or diagnosed with active schistosomiasis, liver cirrhosis or HCC were excluded from the prospective study. All blood samples were collected by one physician, and the same strict protocol was applied to all samples (i.e., centrifugation time and materials). Furthermore, a ‘‘needle to freezer time” of less than 30 min was defined for all samples. All samples were handled at 4°C until they were stored for final analysis at -80°C. RNA extraction and cDNA transcription were performed simultaneously for all samples. The study was approved by the Ethics Committee of Faculty of Pharmacy, Cairo University.

Sixty-seven (49 males and 18 females) consecutive outpatients with ages ranging from 20–55 years with chronic HCV-4 and twenty-seven (17 males and 10 females) healthy volunteers were included in the prospective study. Written informed consent, approved by the Ethics Committee of Faculty of Pharmacy, Cairo University, was obtained from all subjects in this study. Chronic HCV was diagnosed at the Tropical Medicine and Hepatology Department of El-Kasr El Aini Hospital, and patients were referred for antiviral therapy with PEG-IFN/RBV. Blood samples were collected from all patients prior to the start of treatment for analysis of all biochemical markers, HCV viral loads, genotyping, and miRNA estimation. Subsequently, the HCV genotype-4 patients were divided depending on their responses to antiviral therapy into 45 (36 males and 9 females) sustained virological responders (SVR), for whom viral RNA remained undetectable 6 months after the cessation of the 6 month treatment period, and 22 (13 males and 9 females) non-responders (NR), for whom viral RNA remained detectable after 6 months of treatment.

### HCV Genotyping using the Nested-PCR/Ohno Method

RNA was isolated from sera for HCV genotyping using a Qiagen Viral RNA kit (Hilden, Germany). HCV-RNA genotyping was performed using the Ohno method, which relies on nested PCR amplification of the virus core gene using genotype-specific primers as previously described [18].

### Assessment of HCV-4 RNA Loads by Real-Time PCR

Viral loads were measured by real-time reverse transcription polymerase chain reaction (qRT-PCR) using a Light Cycler system (Roche Diagnostics GmbH, Mannheim, Germany) after RNA extraction from sera using a Qiagen Viral RNA kit (Hilden, Germany). Amplification primers for HCV were: 5’ primer K78F (CAAGCACCCTATCAGGCAGT) and 3’ primer K80R (AGCGTCTAGCCATGGCGT). The hybridisation probes FL 5’(GCAGCCTCCAGGACCCCCC)3’ and LC 5’(CCCGGGAGAGCCATAGTGGTCTG)3’ were used to detect the product. HCV-RNA in serum was measured before treatment and routinely at weeks 24 and 48 after treatment and graded into low, moderate and high levels.

### Biochemical Investigations

Blood samples were collected from all volunteers and immediately centrifuged at 4°C; the serum supernatants were frozen at -20°C. All analyses were performed in duplicate. Laboratory tests including ALT, AST, alkaline phosphatase (ALP), total bilirubin (T. Bil), direct bilirubin (D. Bil), albumin (Alb), creatinine and α-fetoprotein (AFP) as well as complete blood counts (CBC) including haemoglobin (Hb) and total leukocyte counts (TLC) were performed in the HCV genotype-4 infected patients treated with PEG-IFN/RBV antiviral therapy and healthy controls.

#### Serum miRNA Assay

Total RNA with preserved miRNAs was extracted from 200 μl serum with the miRNeasy extraction kit (Qiagen, Valencia, CA, USA) using 1 ml QIAzol lysis reagent and incubated for 5 min at RT. Then, 200 μL of chloroform was added, and the samples were vortexed for 15 sec, and incubated for 2–3 min at room temperature. This was followed by centrifugation at 14,000 xg at 4°C for 15 min. The upper watery phase was removed, and an equal volume of 100% ethanol was added. Each 700 μl of this mixture were placed in miRNeasy Mini spin column in a 2 ml collection tube and centrifuged at 8000 xg at room temperature for 15 sec. After the mixture completely passed through the column, 700 μl of buffer RWT was added to each column prior to centrifugation at 8000 xg at room temperature for 15 sec. 500 μl of buffer RPE was added to the column prior to centrifugation at 8000 xg at room temperature for 15 sec. After this, another 500 μl of buffer RPE was added to the column prior to centrifugation at 8000 xg at room temperature for 2 min. The column was placed in a new collection tube and centrifuged at full speed for 2 min. Then, the column was transferred to a new 1.5 ml collection tube, 50 μl of RNase-free water was pipetted directly onto the column and the column was centrifuged for 1 min. at 8000 xg to elute RNA.

Reverse transcription was performed on 5 ng of total RNA in a final volume of 20 μl (incubated for 60 min at 37°C, 5 min at 95°C, and then maintained at 4°C) using the miRNeasy serum/plasma Reverse Transcription Kit (Qiagen, Valencia, CA, USA) according to the manufacturer's instructions.

The expression miR-21, miR-122 and miR-221 were evaluated by qRT-PCR analysis according to the manufacturer's protocol. The housekeeping miRNA SNORD 68 was used as the endogenous control. For real-time PCR, the cDNA template was mixed with SYBER Green Master Mix (Qiagen, Valencia, CA, USA) in a final volume of 25 μl and added to a custom 96-well miScript miRNA PCR array plate enriched with forward and reverse miRNA-specific primers. The plate was sealed with optical thin-wall 8-cap strips. Real-time PCR reactions were performed using an Applied Biosystems 7500 Real Time PCR System (Foster city, CA, USA) with the following conditions: 95°C for 15 min, followed by 40 cycles at 94°C for 15 s, 55°C for 30 s and 70°C for 34 s. The cycle threshold (CT) is defined as the number of cycles required for the fluorescent signal to cross the threshold in real-time PCR. The expression of miRNAs was reported as the ΔCt value, which was calculated by subtracting the CT values of miRNA SNORD68 from the CT values of the target miRNAs. Because there is an inverse correlation between ΔCt and miRNA expression levels, lower ΔCt values are associated with increased miRNA expression. The 2^-ΔΔ (Ct)^ method was used to determine the relative quantitative levels of individual miRNAs.

#### Statistical Analysis

Data are presented as the mean ± standard error (± SE), frequencies (number of cases) and percentages when appropriate. Comparisons of quantitative variables were performed using the nonparametric Kruskal-Wallis test when comparing more than 2 groups and the nonparametric Mann–Whitney U test when comparing 2 groups. HCV PCR results are represented as log HCV PCR. Odds ratios were used to compare the relative odds of the occurrence of the outcome of HCV-4 given exposure to the miRNA expressions markers. Accuracy was represented using the terms sensitivity and specificity. Receiver operator characteristic (ROC) analysis was used to determine the optimum cut-off value for the studied diagnostic markers. P-values less than 0.05 were considered statistically significant.

## Results

### Demographic and laboratory characterisation of the HCV patients and control group

Data represented in Table 1 showed that there was a highly significant difference between the control group and HCV-4 patients in terms of ALT and AST activities, whereas no significant difference in the activities was detected between the NR and SVR patients. ALP activity was significantly higher in the NR patients compared with the normal and SVR subjects. In contrast, TLC was significantly lower in SVR patients compared with normal subjects. The mean values of AFP and log HCV-4 RNA (viral load) were significantly higher in NR compared with SVR patients (p-values = 0.013 and 0.003, respectively).

**Table 1:** Demographic and laboratory characterisation of HCV-4 and control groups.

	Controls	HCV-4 patients	HCV-4 patients	P Value
	N = 27	NR (N = 22)	SVR (N = 45)	
Age	40.89±16.187	40.36±8.488	36.93±7.803	0.263
Females %	37.0%	40.9%	20.0%	
Males %	63.0%	59.1%	80.0%	
ALT (IU/l)	26.74 ± 1.76	66.64 ± 10.84 ^a^	67.93 ± 5.20 ^a^	0.000
AST (IU/l)	21.56 ± 1.46	52.68 ± 8.42 ^a^	53.60 ± 6.52 ^a^	0.001
ALP (IU/l)	65.82 ± 6.39	123.95 ± 17.16^a^ ^c^	87.07 ± 6.40 ^b^	0.001
Alb. (g/dl)	4.02 ± 0.16	4.12 ± 0.07	4.27 ± 0.06	0.162
T. Bil (mg/dl)	0.79 ± 0.06	0.76 ± 0.06	0.70 ± 0.03	0.393
D. Bil (mg/dl)	0.13 ± 0.02	0.17 ± 0.03	0.17 ± 0.02	0.238
AFP (ng/ml)	-	9.00 ± 2.90	3.21 ± 0.49 ^b^	0.013
Log HCV PCR	-	4.89 ± 1.45	3.59 ± 0.24 ^b^	0.003
TLC (10^2^/μl)	7.55 ± 0.41	6.84 ±0.55	5.65 ±0.2045 ^a^	0.001
Hb (g/dl)	13.67 ± 0.2733	13.79 ±0.43	14.34 ± 0.1 8	0.132
Creatinine (mg/dl)	0.93 ± 0.06	0.87 ± 0.03	0.79±0.03 ^a^	0.035

Values are represented as the means ± S.E. NR, non-responders; SVR, sustained viral response; ALT, alanine aminotransferase; AST, aspartate aminotransferase; ALP, alkaline phosphatase, Alb, albumin; T. Bil, total bilirubin; D. Bil, direct bilirubin, AFP, alphafetoprotin; TLC, total leucocyte count; Hg, haemoglobin

^a^ Significant difference from normal control group;

^b^ Significant difference from NR group;

^c^ Significant difference from SVR group.

### miR-122, miR-221, and miR-21 expression levels in HCV-4 patients and normal controls

**Fig 1:**
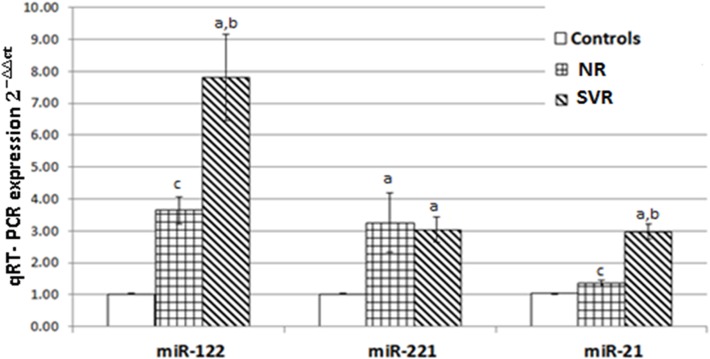
Real-time qPCR of miR-122, miR-221, miR-21 expression levels. Each column represents the relative amount of miRNAs normalised to the expression of the normal control. The data shown are mean ± SE. of the three independent experiments. a: indicates a significant difference from the normal control group; b: indicates a significant difference from NR; c: indicates a significant difference from SVR at P < 0.05.

The data presented in Fig. 1 demonstrate that there was a highly significant increase in the quantitative expression levels of miR-122, miR-221 and miR-21 in all HCV-4 patients compared with the normal control group (p-value <0.05). However, although there was no significant difference in miR-221 quantitative expression between the NR and SVR groups, there was a significant difference in the quantitative expression of miR-21 and miR-122.

### Correlation between miR-122, miR-221 and miR-21 and viral load

**Fig 2:**
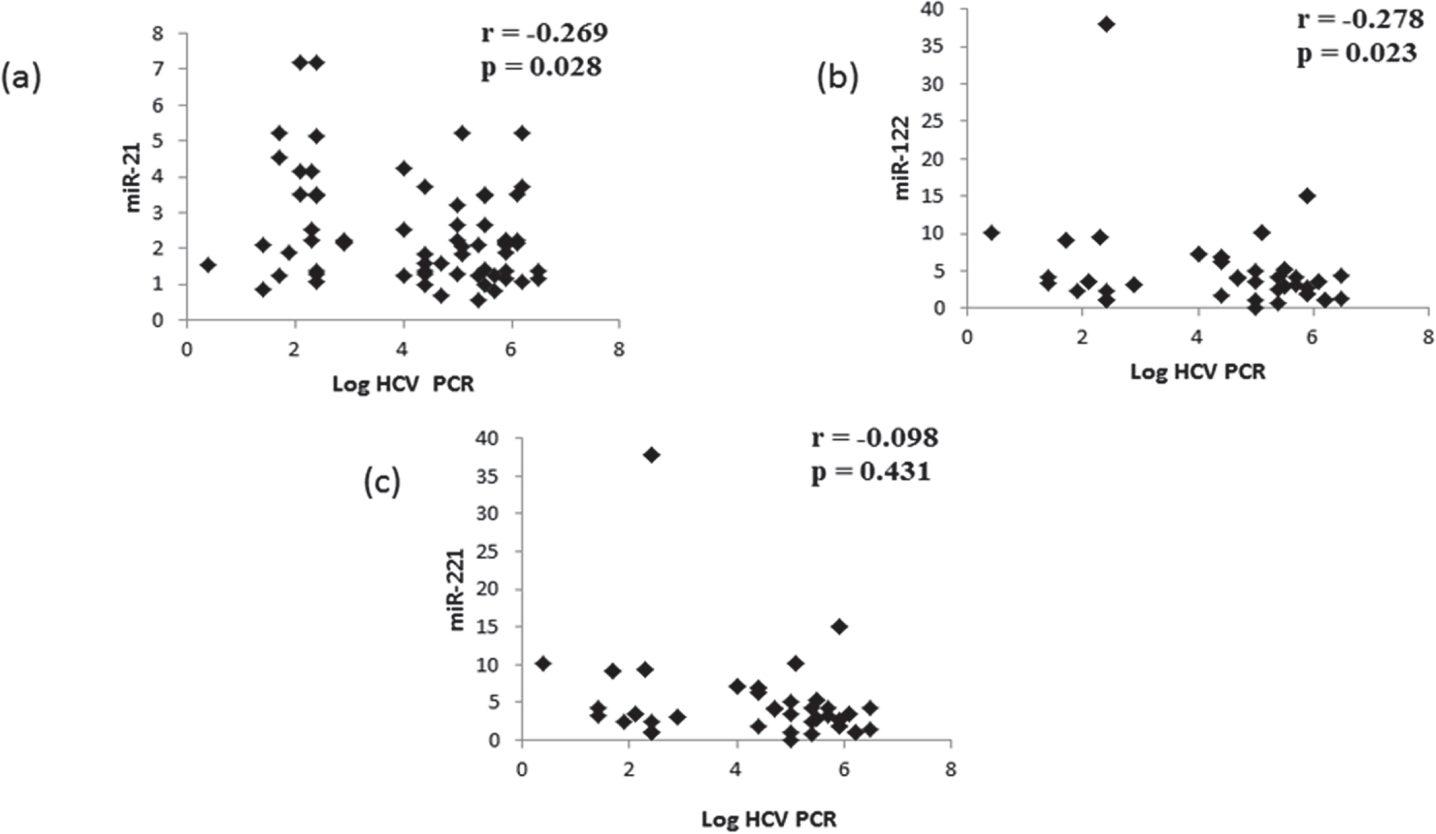
Correlation between log HCV PCR and miR-21 (a), miR-122 (b), and miR-221 (c) in patients with HCV-4. Points represent 2^−ΔΔt^ values for miRNAs normalised to normal controls. Difference was considered significant at P < 0.05.

To further verify the correlation between the expression levels of miR-122, miR-221 and miR-21 with viral load among the HCV-4 cases, multivariate logistic regression analysis with Waldʼs test was used. As shown in Fig. 2, log HCV PCR showed a significant inverse correlation with miR-21 and miR-122 quantitative expression levels in HCV-4 patients. However, there was no significant correlation between log HCV PCR and miR-221 quantitative expression levels despite the highly significant difference between the control group and HCV-4 patients in the mean value of miRNA-221.

### Measurement of the power of miR-122, miR-221 and miR-21 to predict drug responses in HCV-4 patients

**Fig 3:**
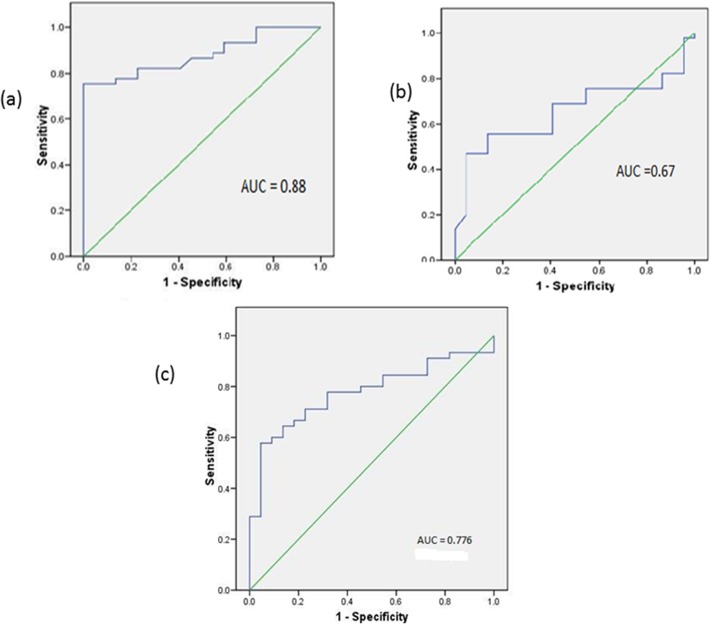
Receiver operator characteristic (ROC) curve analysis displaying the diagnostic power in predicting SVR in HCV-4 patients when analysed as a single marker for (a) miR-21 and (b) miR-122, or as combined markers (c) miR-21 and miR-122.

Multiple logistic regression analysis was performed to determine whether the miRNA markers could predict the drug response in HCV-4 patients. Table 2 showed that miR-21 was an independent predictor for the drug response, with an odds ratio of 1.68 (p = 0.002). Receiver operator characteristic (ROC) analysis was used to determine the optimum cut-off value for the studied diagnostic markers. Fig. 3 represents a ROC curve for the prediction of the drug response among HCV-4 cases by the quantitative expression of miR-21 and miR-221. The sensitivity and specificity of miR-21 calculated in this study were 82.2% and 77.3%, respectively, with a cut-off value of 1.7 and a positive predictive value of 88.1%. The sensitivity and specificity of miR-122 were 68.9% and 59.1%, respectively, with a cut-off value of 3.5 and a positive predictive value of 77.5%. Finally, the sensitivity and specificity of combined miR-21 and miR-122 quantitative expression calculated in this study were 55.6% and 95.5%, respectively, whereas the positive predictive value was 96.2%.

**Table 2:** Logistic regression analysis showing the association of miR-122, miR-221 and miR-21 with drug responsiveness among HCV-4 patients.

	**Odds ratio**	**95%CI for OR**	**P value**
**miR-122**	1.020	0.933–1.115	0.106
**miR-221**	0.967	0.831–1.124	0.659
**miR-21**	1.681	1.201–2.354	0.002*

* Difference was considered significant at P < 0.05.

## Discussion

Predictors of response serve as decision-making tools and help treating physicians identify patients who are likely or unlikely to achieve SVR. Thus, these measures reduce the risk of side effects and cost, and spare patients the disappointment of treatment failure [19]. In the present study, we analysed several factors that are associated with HCV infection and are known to contribute to disease prognosis and response to treatment. These factors are gender, age, liver enzyme levels (ALT and AST), AFP, and HCV-RNA levels (viral load). ALT, AST, and AFP levels were significantly increased in HCV-4 infected patients in comparison to healthy controls. Serum AFP levels significantly differed between NR and SVR patients. This finding is in agreement with a previous study that included 100 Egyptian patients with chronic hepatitis C [20], and suggests that serum AFP levels should be added to the list of predictive factors for treatment responses. Backus et al. [21] identified 5944 hepatitis C patients who were treated at Veterans Affairs Health Care with PEG-IFN, and found that patients with low viremia were more likely to respond compared with patients with a high viral load. Similarly, our study confirmed low viremia as a predictor of response to therapy. Recently, direct-acting antiviral agents (DAAs) were added to PEG-IFN/RBV, leading to higher sustained response rates in genotype 1 infected individuals. DAAs are directed toward specific proteins involved in hepatitis C replication, with the NS3/NS4A protease inhibitors the furthest in development. Telaprevir and boceprevir are NS3/NS4a inhibitors that significantly improve sustained responses when added to PEG-IFN/RBV [22]. In addition to improving SVR rates in HCV genotype-1 treatment-naïve patients, these drugs administered as a triple combination with PEG-IFN/RBV provide opportunities for more patients to receive a shorter durations of treatment (24–28 weeks) than with PEG-IFN/RBV alone (48 weeks) [23].

MicroRNAs represent an interesting field of investigation with regards HCV infection and replication. Furthermore, they may represent new targets for the development of antiviral therapeutics [24]. miRNA levels have been reported to differ depending on a patient’s drug response. As a result, miRNAs can be useful for predicting patient drug responses before the administration of combination therapy, thereby reducing ineffective treatments.

The present study demonstrated that HCV-4 patients’ serum samples showed significantly higher expression levels of miR-21, miR-122, and miR-221 than the normal controls. This finding suggests that all three miRNAs may be useful as diagnostic biomarkers for the diagnosis of HCV-4. The up-regulation in the expression level of miR-21 in HCV-4 patients could be due to the increased proliferation of liver cells during HCV infection and/or during the late stages of fibrosis [25]. Similar to the present data, it has recently been reported that sera from HBV-induced chronic hepatitis patients contain elevated levels of miR-21 in comparison to healthy controls [26]. However, in another study with patients suffering from HBV-induced chronic hepatitis, no elevation of serum miR-21 levels was found despite strongly elevated ALT levels in these patients [27].

In our study, the liver-specific microRNA miR-122 was upregulated in HCV-4 patients compared with normal controls. miR-122 is known to be a positive cofactor in the HCV replication cycle [28] and serves as a biomarker for hepatic injury [29]. miR-122 facilitates the replication of HCV in both host hepatic cells [30] and non-hepatic cells [31]. However, the mechanism by which miR-122 regulates HCV is not fully understood. miR-122 does not directly affect HCV RNA synthesis in cells or in isolated replication complexes [32]. Previous work has shown that miR-122 binding to the HCV 5'UTR stimulates HCV internal ribosome entry site (IRES)-driven translation [33, 34]. Recently, miR-122 binding was shown to stabilise HCV RNA by protecting it from degradation by the 5'-3' exonuclease Xrn1, and it was proposed that previous observations of the activation of translation by miR-122 could instead be explained by RNA stabilisation [35].

The profound upregulation of miR-221 in HCV-4 patients could be an indicator of the increased susceptibility of patients to hepatocellular carcinoma (HCC), because this miRNA has been implicated as a contributor to liver tumourigenesis [36]. Indeed, miR-221 has been proposed as a potential target for therapeutic intervention in HCC and fulminant liver failure [37, 38]. It is important to note that miR-221 up-regulation has commonly been associated with advanced tumour stages, lower survival rates, and higher recurrence rates in human HCC [39].

Data obtained from this study showed that patients with SVR following pegIFN/RBV therapy had significantly higher pretreatment serum miR-21 and miR-122 levels than NR patients. The same observation concerning miR-122 was reported by [40] and verified by [41], who revealed that HCV-infected patients who did not respond to therapy had significantly lower miR-122 levels compared with responders. A recent study revealed that the mammalian liver can utilise cellular miRNAs, especially miRNA-122, to fight viral infections through the IFN system [13]. In contrast, an *in vivo* study showed that there was no correlation between miR-122 expression and the HCV load in chronic hepatitis patients undergoing IFN therapy [42]. Multiple logistic regression analyses revealed that serum miR-21 and miR-122 levels were correlated with log HCV PCR in HCV-4 patients treated with PEG-IFN /RBV. To the best of our knowledge, this is the first report to correlate miR-21 with the response to PEG-IFN/RBV in HCV-4 patients. This finding suggests that higher pretreatment serum miR-21 and miR-122 levels might predict favourable virological responses to PEG-IFN/RBV therapy in patients with HCV-4. To quantify how strongly miR-21 and miR-122 levels predict the PEG-IFN/RBV therapy response in HCV-4 patients, we performed an OR test. The OR test revealed that miR-21 has a higher predictive value than miR-122 in differentiating NR from SVR patients.

The diagnostic performance of miR-21, miR-122, and a combination of these markers in differentiating NR from SVR patients was evaluated by ROC curve analysis. The ROC curve of miR-21 reflected a strong separation between NR and SVR patients, with an AUC of 0.88. The ROC curve of miR-122 showed a moderate ability to distinguish between NR and SVR patients, with an AUC of 0.67. In contrast, miR-221 showed a poor ability to distinguish between NR and SVR patients. The combination of miR-21 and miR-122 strongly differentiated between NR and SVR patients, with an AUC of 0.776 and sensitivity, specificity and positive predictive values of 57.8, 95.5 and 96.2%, respectively, with a cut-off value of 3.9.

In conclusion, the present study demonstrated that circulating extracellular miR-21 and miR-122 levels in serum samples from HCV-4 patients can be used to differentiate between the responses of NR and SVR to PEG-IFN/RBV therapy. Moreover, miR-21 appears to have a higher predictive response power than miR-122.
